# Pyrexia of Unknown Origin: A Report of Two Cases

**DOI:** 10.7759/cureus.54059

**Published:** 2024-02-12

**Authors:** Rohan Raj, Parvinder Kaur, Rachel A O’dare, Amarjot S Sandhu, Beegum Tasneem, Beegam Sulthan, Malavika Jayan, Satvir Singh

**Affiliations:** 1 Internal Medicine, Nalanda Medical College and Hospital, Patna, IND; 2 Internal Medicine, Crimean State Medical University, Simferopol, UKR; 3 Nursing, South University, Savannah, USA; 4 General Medicine, Medical University Graz, Graz, AUT; 5 Internal Medicine, Sri Guru Ram Das Institute of Medical Sciences and Research, Sri Amritsar, IND; 6 Internal Medicine, College of Medicine, Ras Al-Khaimah (RAK) Medical and Health Sciences University, Ras Al-Khaimah, ARE; 7 Internal Medicine, Azeezia Institute of Medical Science and Research, Kollam, IND; 8 Internal Medicine, Bangalore Medical College and Research Institute, Bangalore, IND; 9 Internal Medicine, Good Samaritan Hospital, Bakersfield, USA

**Keywords:** non-hodgkin's lymphoma, pyrexia, splenic tuberculosis, pyrexia of unknown origin, fever of unknown origin

## Abstract

Pyrexia of unknown origin (PUO) is a prolonged fever lasting several weeks without an identifiable cause despite extensive medical evaluation. Many a time, its cause remains largely unknown even after collecting a detailed medical history, conducting comprehensive physical assessments, and performing various standard laboratory tests and imaging procedures. This case series presents two cases of pyrexia of unknown origin. The first case includes a unique and uncommon presentation of non-Hodgkin's lymphoma. In the second case, the patient's fever remained unexplained after various investigations and treatments. The two documented cases of PUO presented in this report aim to contribute to the understanding of its diverse etiology and diagnostic challenges. By highlighting unique presentations and diagnostic dilemmas, the cases aim to promote awareness and facilitate timely recognition and appropriate management of PUO.

## Introduction

Pyrexia of unknown origin (PUO) was originally defined in 1961 by Petersdorf and Beeson as a condition where the body temperature exceeds 38.3°C on at least three instances over a period of at least three weeks, with no diagnosis made despite one week of inpatient investigation [1]. Despite the fact that clinicians often categorize the diagnosis of PUO into five common groups (infections, neoplasms, noninfectious inflammatory disorders, miscellaneous conditions, and undiagnosed illnesses), there is currently no consensus on a standardized set of classifications for PUO diseases [2]. In developing nations, infections constitute the primary etiology of PUO, while non-infectious inflammatory diseases (NIID) predominate in industrialized nations. Most patients with PUO exhibit rare symptoms of a common disease rather than common symptoms of a rare disease [3]. Patients with PUOs have varying outcomes based on the concomitant illnesses and underlying diagnosis. Studies on PUO published since 1990 have shown a range of rates of no diagnosis, varying between 9% and 51%. The outcome of patients with PUO depends upon the underlying diagnosis and the presence of comorbid conditions [4]. Most adults who remain undiagnosed even after an extensive evaluation generally have a good prognosis. The use of empirical therapy should be limited to individuals who additionally present with clinical deterioration, neutropenic fever, giant cell arteritis, or suspected life-threatening underlying causes [5]. Herein, we present two cases that highlight the complexity of diagnosing PUO and the need for comprehensive evaluation and multidisciplinary management.

## Case presentation

Case 1

A 60-year-old male patient with no known comorbidities presented with a six-month history of persistent undiagnosed fever, substantial weight loss, splenomegaly, and generalized weakness. The patient had undergone investigations and treatment at a local health center, where his illness was misdiagnosed to be that of an infection. He was started on multiple courses of antibiotics, with no improvement in symptoms.

**Figure 1:**
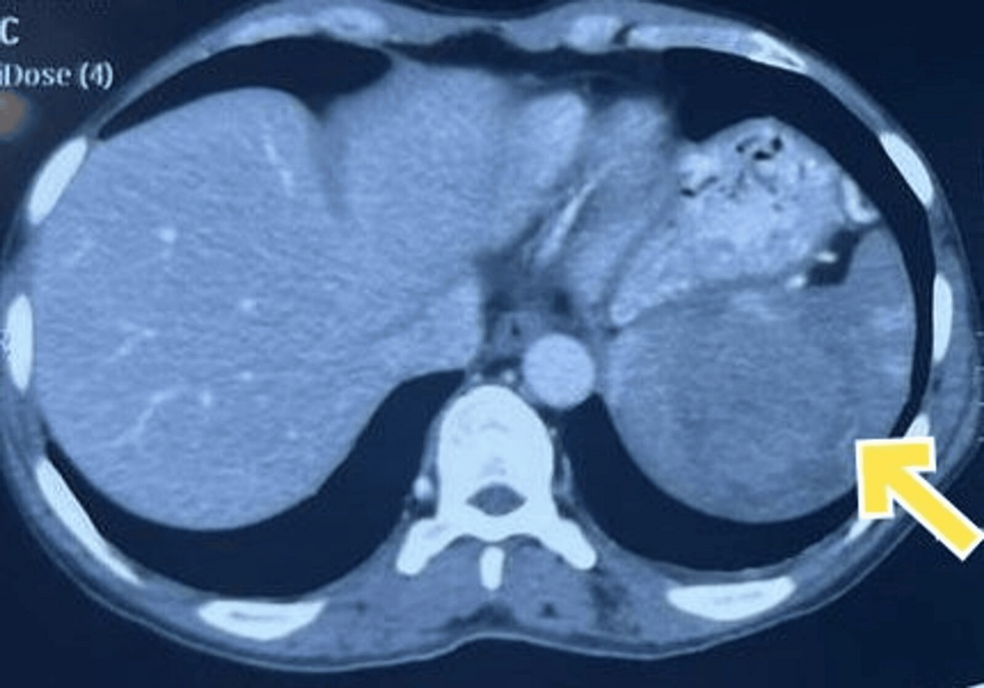
CT image showing an isolated mass-like lesion in the spleen

Upon his visit to our center, the patient exhibited significant weakness, was unable to walk, and had persistent fever. Routine blood and urine investigations were unremarkable. Further investigation through a computed tomography (CT) scan revealed an isolated mass-like lesion in the spleen, as shown in Figure 1. Biopsy of the splenic lesion later yielded the final diagnosis of splenic non-Hodgkin's lymphoma (NHL) of B-cell origin, which was made on the basis of microscopy and immunohistochemistry (CD45 and CD20 positive). Notably, there was no involvement of other organs. The patient was started on CHOP-based chemotherapy (Cyclophosphamide, Hydroxydaunorubicin, Oncovin, and Prednisone) and is currently being followed up.

Case 2

A 29-year-old P1L1 female patient presented to our center with a persistent low-grade fever of about 38°C for five months. Her fever remained unrelenting despite multiple courses of injectable antibiotics provided at a different center. She had no history of cough with expectoration, evening rise of temperature, weight loss, or immunosuppressant drug intake.

**Figure 2:**
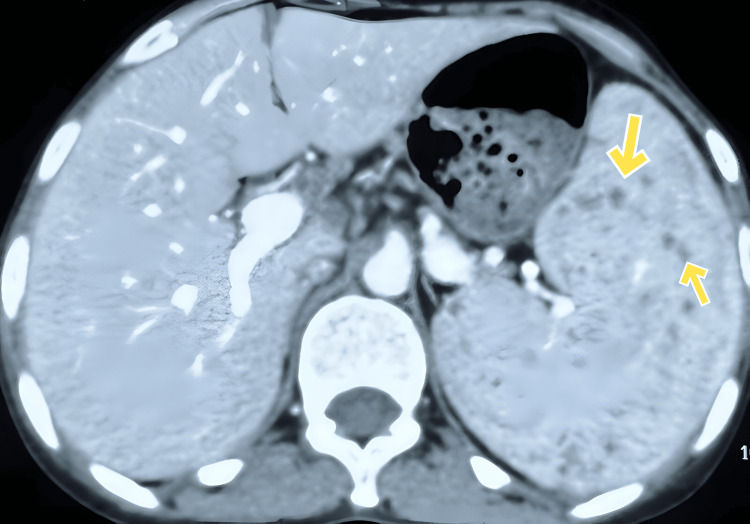
CT image showing the presence of multiple micro-abscesses in the spleen

Upon her admission to our center, routine blood and urine investigations were conducted, and the results were unremarkable. The Tuberculin skin test (Mantoux test) yielded a negative result. A CT abdomen was later conducted, which revealed the presence of multiple micro-abscesses in the spleen, without any indication of abscess formation in adjacent areas, as shown in Figure 2. Based on these radiological findings and considering her origin in a tuberculosis-endemic region, a suspected diagnosis of splenic tuberculosis was made. Following this, a fine needle aspiration cytology (FNAC) was performed to obtain a definitive diagnostic confirmation. FNAC revealed caseating granulomas and was positive for acid-fast bacilli (AFB). The patient was then started on the standard ATT regimen consisting of Isoniazid (INH), Rifampicin (RIF), Ethambutol (EMB), and Pyrazinamide (PZA) for a period of six months and is currently being followed up.

## Discussion

Despite several years after the first definition of pyrexia of unknown origin, it still remains a diagnostic challenge. Its main diagnostic evaluation is by identifying potential diagnostic clues (PDCs), which take into account the signs, symptoms, and abnormalities that point to a specific condition. Proper history taking as well as the analysis of risk factors are also warranted [3].

Non-invasive diagnostic assessments encompass a range of tests such as complete blood count, urinalysis, erythrocyte sedimentation rate (ESR), C-reactive protein (CRP), lactate dehydrogenase (LDH), tests for typhoid and Brucellosis, chest x-ray, abdominal and pelvic CT scans and antinuclear antibody (ANA) titration. Invasive tests include biopsies of lymph nodes, liver, bone marrow, epididymal nodule, and temporal artery. Endoscopic examinations are done while suspecting Crohn's disease and gastrointestinal tumors [6].

The first case of splenic involvement in NHL presented in this case series highlights the significance of considering lymphoproliferative disorders in the differential diagnosis of splenomegaly and pyrexia of unknown origin. Splenic NHL is characterized by an infiltration of small to medium-sized lymphocytes, plasma cells, and monocytoid B-cells, not attributed to another primary cause [7]. Clinical features aiding in its diagnosis include fever, sweating, weight loss, and left upper quadrant pain. During lab evaluation, leukocytosis, anemia, an increased ESR, and an elevated LDH are often observed. Observation of splenomegaly on radiological assessment and pathological evaluation following biopsy are also important. Treatment modalities generally include a mixture of radiation, chemotherapy, and monoclonal antibodies [8].

The second case of splenic tuberculosis highlights the importance of considering infectious etiologies in patients presenting with pyrexia of unknown origin and splenic involvement. Splenic tuberculosis is generally seen as an opportunistic infection in immunocompromised hosts, or as a part of disseminated pulmonary tuberculosis [9]. In a case report of eight patients with splenic tuberculosis, abdominal CT revealed multiple hypodense regions in the spleen, with two patients having multiple abscesses. Invasive tests that followed in these two patients included aspiration cytology of the splenic lesion, revealing caseating granulomas in one, and percutaneous drainage of the splenic abscess in the other. Later, microscopic examinations for AFB and AFB cultures were performed. All the patients underwent a splenectomy. Gross specimens showed multiple caseating nodules and enlarged lymph nodes while histopathological examination revealed epithelioid granulomas with central caseous necrosis, suggesting splenic tuberculosis [10]. Treatment modalities for splenic tuberculosis include percutaneous aspiration or splenectomy. This is followed by anti-tubercular treatment, consisting of Isoniazid, Rifampicin, Ethambutol, and Pyrazinamide. In some cases, anti-tubercular treatment alone is prescribed [11].

## Conclusions

In conclusion, PUO is a challenging clinical entity with a diverse etiology necessitating a systematic diagnostic approach. Timely recognition and appropriate management are crucial to prevent potential complications and improve patient outcomes. This first case presents a rare manifestation of NHL, emphasizing the importance of considering unusual presentations in patients with PUO. The isolated splenic involvement in the absence of organ spread makes it an atypical case, requiring comprehensive evaluation and a multidisciplinary approach. The second case highlights the challenging nature of diagnosing PUO and the importance of thorough investigations. The presence of splenic tuberculosis, as suggested by the imaging findings, underscores the need for further confirmation through invasive procedures such as aspiration and FNAC.
